# Supplementary material for: Shift work and long work hours and their association with chronic health conditions: A systematic review of systematic reviews with meta-analyses
Source: PLoS One. 2020 Apr 2;15(4):e0231037. doi: 10.1371/journal.pone.0231037 (PMC7117719; doi:10.1371/journal.pone.0231037)
Supplement: S1 File — (PDF) [file pone.0231037.s001.pdf]

PubMed MEDLINE

(("Shift Work Schedule"[Mesh] OR shift work*[tiab] OR shiftwork*[tiab] OR evening shift*[tiab] OR night shift*[tiab] OR night-shift[tiab] OR day shift*[tiab] OR day-shift*[tiab] OR night work*[tiab] OR evening work*[tiab] OR split shift*[tiab] OR split-shift*[tiab] OR rotating shift*[tiab] OR rotating-shift*[tiab] OR alternating work*[tiab] OR alternating-work*[tiab] OR non day work*[tiab] OR non-day-work*[tiab] OR "work hours"[tiab] OR "working hours"[tiab] OR "overtime work"[tiab] OR "extended work"[tiab] OR "extended shift"[tiab] OR "long work"[tiab] OR "long working"[tiab] OR "long shift"[tiab] OR "12-hour shift"[tiab] OR "12-hour work day"[tiab] OR "compressed work"[tiab] OR "four-day week"[tiab] OR compressed shift*[tiab] OR "rearranged work week"[tiab] OR shift length*[tiab] OR "shift duration"[tiab] OR "long hours"[tiab] OR "work schedule tolerance"[mesh]))

AND (systematic[sb] OR meta-analysis[pt] OR meta-analysis as topic[mh] OR meta-analysis[mh] OR meta analy*[tw] OR metanaly*[tw] OR metaanaly*[tw] OR met analy*[tw] OR integrative research[tiab] OR integrative review*[tiab] OR integrative overview*[tiab] OR research integration*[tiab] OR research overview*[tiab] OR collaborative review*[tiab] OR collaborative overview*[tiab] OR systematic review*[tiab] OR technology assessment*[tiab] OR technology overview*[tiab] OR "Technology Assessment, Biomedical"[mh] OR HTA[tiab] OR HTAs[tiab] OR comparative efficacy[tiab] OR comparative effectiveness[tiab] OR outcomes research[tiab] OR indirect comparison*[tiab] OR ((indirect treatment[tiab] OR mixed-treatment[tiab]) AND comparison*[tiab]) OR Embase*[tiab] OR Cinahl*[tiab] OR systematic overview*[tiab] OR methodological overview*[tiab] OR methodologic overview*[tiab] OR methodological review*[tiab] OR methodologic review*[tiab] OR quantitative review*[tiab] OR quantitative overview*[tiab] OR quantitative synthes*[tiab] OR pooled analy*[tiab] OR Cochrane[tiab] OR Medline[tiab] OR Pubmed[tiab] OR Medlars[tiab] OR handsearch*[tiab] OR hand search*[tiab] OR meta-regression*[tiab] OR metaregression*[tiab] OR data synthes*[tiab] OR data extraction[tiab] OR data abstraction*[tiab] OR mantel haenszel[tiab] OR peto[tiab] OR der-simonian[tiab] OR dersimonian[tiab] OR fixed effect*[tiab] OR "Cochrane Database Syst Rev"[Journal:__jrid21711] OR "health technology assessment winchester, england"[Journal] OR "Evid Rep Technol Assess (Full Rep)"[Journal] OR "Evid Rep Technol Assess (Summ)"[Journal] OR "Int J Technol Assess Health Care"[Journal] OR "GMS Health Technol Assess"[Journal] OR "Health Technol Assess (Rockv)"[Journal] OR "Health Technol Assess Rep"[Journal])

Embase.com

'shift work'/exp OR 'shift worker'/exp OR 'shift work*':ti,ab OR shiftwork*:ti,ab OR 'evening shift*':ti,ab OR 'night shift*':ti,ab OR 'night shift':ti,ab OR 'day shift*':ti,ab OR 'night work*':ti,ab OR 'evening work*':ti,ab OR 'split shift*':ti,ab OR 'rotating shift*':ti,ab OR 'alternating work*':ti,ab OR 'non day work*':ti,ab OR 'work hours':ti,ab OR 'working hours':ti,ab OR 'overtime work':ti,ab OR 'extended work':ti,ab OR 'extended shift':ti,ab OR 'long work':ti,ab OR 'long working':ti,ab OR 'long shift':ti,ab OR '12-hour shift':ti,ab OR '12-hour work day':ti,ab OR 'compressed work':ti,ab OR 'four-day week':ti,ab OR 'compressed shift*':ti,ab OR 'rearranged work week':ti,ab OR 'shift length*':ti,ab OR 'shift duration':ti,ab OR 'long hours':ti,ab OR 'work schedule'/exp

AND

'meta analysis'/exp OR 'systematic review'/exp OR (meta NEAR/3 analy*):ab,ti OR metaanaly*:ab,ti OR review*:ti OR overview*:ti OR (synthes* NEAR/3 (literature* OR research* OR studies OR data)):ab,ti OR (pooled AND analys*:ab,ti) OR ((data NEAR/2 pool*):ab,ti AND studies:ab,ti) OR medline:ab,ti OR medlars:ab,ti OR embase:ab,ti OR cinahl:ab,ti OR scisearch:ab,ti OR psychinfo:ab,ti OR psycinfo:ab,ti OR psychlit:ab,ti OR psyclit:ab,ti OR cinhal:ab,ti OR cancerlit:ab,ti OR cochrane:ab,ti OR bids:ab,ti OR pubmed:ab,ti OR ovid:ab,ti OR ((hand OR manual OR database* OR computer*) NEAR/2 search*):ab,ti OR (electronic NEAR/2 (database* OR 'data base' OR 'data bases')):ab,ti OR bibliograph*:ab OR 'relevant journals':ab OR ((review* OR overview*) NEAR/10 (systematic* OR methodologic* OR quantitativ* OR research* OR literature* OR studies OR trial* OR effective*)):ab NOT (((retrospective* OR record* OR case* OR patient*) NEAR/2 review*):ab,ti OR ((patient* OR review*) NEAR/2 chart*):ab,ti OR rat:ab,ti OR rats:ab,ti OR mouse:ab,ti OR mice:ab,ti OR hamster:ab,ti OR hamsters:ab,ti OR animal:ab,ti OR animals:ab,ti OR dog:ab,ti OR dogs:ab,ti OR cat:ab,ti OR cats:ab,ti OR bovine:ab,ti OR sheep:ab,ti) NOT ('editorial'/exp OR 'erratum'/de OR 'letter'/exp) NOT ('animal'/exp OR 'nonhuman'/exp NOT ('animal'/exp OR 'nonhuman'/exp AND 'human'/exp))

CINAHL (Ebsco.com)

((MH "Shiftwork") OR (MH "Shift Workers")) OR TI (‘shift work*’ OR shiftwork* OR ‘evening shift*’ OR ‘night shift*’ OR night-shift OR ‘day shift*’ OR day-shift* OR ‘night work*’ OR ‘evening work*’ OR ‘split shift*’ OR split-shift* OR ‘rotating shift*’ OR rotating-shift* OR ‘alternating work*’ OR alternating-work* OR ‘non day work*’ OR non-day-work* OR ‘work hours’ OR ‘working hours’ OR ‘overtime work’ OR ‘extended work’ OR ‘extended shift’ OR ‘long work’ OR ‘long working’ OR ‘long shift’ OR ‘12-hour shift’ OR ‘12-hour work day’ OR ‘compressed work’ OR ‘four-day week’ OR ‘compressed shift*’ OR ‘rearranged work week’ OR ‘shift length*’ OR ‘shift duration’ OR ‘long hours’) OR AB (‘shift work*’ OR shiftwork* OR ‘evening shift*’ OR ‘night shift*’ OR night-shift OR ‘day shift*’ OR day-shift* OR ‘night work*’ OR ‘evening work*’ OR ‘split shift*’ OR split-shift* OR ‘rotating shift*’ OR rotating-shift* OR ‘alternating work*’ OR alternating-work* OR ‘non day work*’ OR non-day-work* OR ‘work hours’ OR ‘working hours’ OR ‘overtime work’ OR ‘extended work’ OR ‘extended shift’ OR ‘long work’ OR ‘long working’ OR ‘long shift’ OR ‘12-hour shift’ OR ‘12-hour work day’ OR ‘compressed work’ OR ‘four-day week’ OR ‘compressed shift*’ OR ‘rearranged work week’ OR ‘shift length*’ OR ‘shift duration’ OR ‘long hours’)

AND

(MH "Meta Analysis") OR TI meta analys* OR AB meta analys* OR TI Metaanaly* OR AB metaanalys* OR (MH "Literature Review+") OR TI systematic review* OR AB systematic review* OR TI systematic overview* OR AB systematic overview* NOT (PT commentary OR PT letter OR PT editorial OR MH animals+)

Web of Science

TOPIC: (“shift work*” OR shiftwork* OR “evening shift*” OR “night shift*” OR “night-shift” OR “day shift*” OR “day-shift*” OR “night work*” OR “evening work*” OR “split shift*” OR “split-shift*” OR “rotating shift*” OR “rotating-shift*” OR “alternating work*” OR “alternating-work*” OR “non day work*” OR “non-day-work*” OR “work hours” OR “working hours” OR “overtime work” OR “extended work” OR “extended shift” OR “long work” OR “long working” OR “long shift” OR “12-hour shift” OR “12-

hour work day" OR "compressed work" OR "four-day week" OR "compressed shift*" OR "rearranged work week" OR "shift length*" OR "shift duration" OR "long hours")

AND

TOPIC: (("meta analy*" OR metanaly* OR metaanaly* OR "met analy*" OR "integrative research" OR "integrative review*" OR "integrative overview*" OR "research integration*" OR "research overview*" OR "collaborative review*" OR "collaborative overview*" OR "systematic review*" OR "technology assessment*" OR "technology overview*" OR HTA OR HTAs OR "comparative efficacy" OR "comparative effectiveness" OR "outcomes research" OR "indirect comparison*" OR (("indirect treatment" OR mixed-treatment) AND comparison*) OR Embase* OR Cinahl* OR "systematic overview*" OR "methodological overview*" OR "methodologic overview*" OR "methodological review*" OR "methodologic review*" OR "quantitative review*" OR "quantitative overview*" OR "quantitative synthes*" OR "pooled analy*" OR Cochrane OR Medline OR Pubmed OR Medlars OR handsearch* OR hand search* OR meta-regression* OR metaregression* OR "data synthes*" OR "data extraction" OR "data abstraction*" OR "mantel haenszel" OR peto OR der-simonian OR dersimonian OR "fixed effect*"))

- Exclude letters, editorials, news

Scopus

(TITLE-ABS-KEY ("shift work*" OR shiftwork* OR "evening shift*" OR "night shift*" OR night-shift OR "day shift*" OR day-shift* OR "night work*" OR "evening work*" OR "split shift*" OR split-shift* OR "rotating shift*" OR rotating-shift* OR "alternating work*" OR alternating-work* OR "non day work*" OR non-day-work* OR "work hours" OR "working hours" OR "overtime work" OR "extended work" OR "extended shift" OR "long work" OR "long working" OR "long shift" OR "12-hour shift" OR "12-hour work day" OR "compressed work" OR "four-day week" OR "compressed shift*" OR "rearranged work week" OR "shift length*" OR "shift duration" OR "long hours")) AND (((TITLE-ABS-KEY ("shift work*" OR shiftwork* OR "evening shift*" OR "night shift*" OR night-shift OR "day shift*" OR day-shift* OR "night work*" OR "evening work*" OR "split shift*" OR split-shift* OR "rotating shift*" OR rotating-shift* OR "alternating work*" OR alternating-work* OR "non day work*" OR non-day-work* OR "work hours" OR "working hours" OR "overtime work" OR "extended work" OR "extended shift" OR "long work" OR "long working" OR "long shift" OR "12-hour shift" OR "12-hour work day" OR "compressed work" OR "four-day week" OR "compressed shift*" OR "rearranged work week" OR "shift length*" OR "shift duration" OR "long hours"))) AND (TITLE-ABS-KEY ((meta AND analy*) OR metanaly* OR metaanaly* OR (met AND analy*) OR "integrative research" OR "integrative review*" OR "integrative overview*" OR "research integration*" OR "research overview*" OR "collaborative review*" OR "collaborative overview*" OR "systematic review*" OR "technology assessment*" OR "technology overview*" OR hta OR htas OR "comparative efficacy" OR "comparative effectiveness" OR "outcomes research" OR "indirect comparison*" OR (("indirect treatment" OR mixed-treatment) AND comparison*) OR embase* OR cinahl* OR "systematic

overview*" OR "methodological overview*" OR "methodologic overview*" OR "methodological review*" OR "methodologic review*" OR "quantitative review*" OR "quantitative overview*" OR "quantitative synthes*" OR "pooled analy*" OR cochrane OR medline OR pubmed OR medlars OR handsearch* OR (hand AND search*) OR meta-regression* OR metaregression* OR "data synthes*" OR "data extraction" OR "data abstraction*" OR "mantel haenszel" OR peto OR der-simonian OR dersimonian OR "fixed effect*")) **)**

PsycINFO

DE "Workday Shifts" OR DE "Work Scheduling" OR TI ('shift work*' OR shiftwork* OR 'evening shift*' OR 'night shift*' OR night-shift OR 'day shift*' OR day-shift* OR 'night work*' OR 'evening work*' OR 'split shift*' OR split-shift* OR 'rotating shift*' OR rotating-shift* OR 'alternating work*' OR alternating-work* OR 'non day work*' OR non-day-work* OR 'work hours' OR 'working hours' OR 'overtime work' OR 'extended work' OR 'extended shift' OR 'long work' OR 'long working' OR 'long shift' OR '12-hour shift' OR '12-hour work day' OR 'compressed work' OR 'four-day week' OR 'compressed shift*' OR 'rearranged work week' OR 'shift length*' OR 'shift duration' OR 'long hours') OR AB ('shift work*' OR shiftwork* OR 'evening shift*' OR 'night shift*' OR night-shift OR 'day shift*' OR day-shift* OR 'night work*' OR 'evening work*' OR 'split shift*' OR split-shift* OR 'rotating shift*' OR rotating-shift* OR 'alternating work*' OR alternating-work* OR 'non day work*' OR non-day-work* OR 'work hours' OR 'working hours' OR 'overtime work' OR 'extended work' OR 'extended shift' OR 'long work' OR 'long working' OR 'long shift' OR '12-hour shift' OR '12-hour work day' OR 'compressed work' OR 'four-day week' OR 'compressed shift*' OR 'rearranged work week' OR 'shift length*' OR 'shift duration' OR 'long hours')

AND

DE "Meta Analysis" OR TI ("meta analy*" OR metanaly* OR metaanaly* OR "met analy*" OR "integrative research" OR "integrative review*" OR "integrative overview*" OR "research integration*" OR "research overview*" OR "collaborative review*" OR "collaborative overview*" OR "systematic review*" OR "technology assessment*" OR "technology overview*" OR HTA OR HTAs OR "comparative efficacy" OR "comparative effectiveness" OR "outcomes research" OR "indirect comparison*" OR (("indirect treatment" OR mixed-treatment) AND comparison*) OR Embase* OR Cinahl* OR "systematic overview*" OR "methodological overview*" OR "methodologic overview*" OR "methodological review*" OR "methodologic review*" OR "quantitative review*" OR "quantitative overview*" OR "quantitative synthes*" OR "pooled analy*" OR Cochrane OR Medline OR Pubmed OR Medlars OR handsearch* OR hand search* OR meta-regression* OR metaregression* OR "data synthes*" OR "data extraction" OR "data abstraction*" OR "mantel haenszel" OR peto OR der-simonian OR dersimonian OR "fixed effect*") OR AB ("meta analy*" OR metanaly* OR metaanaly* OR "met analy*" OR "integrative research" OR "integrative review*" OR "integrative overview*" OR "research integration*" OR "research overview*" OR "collaborative review*" OR "collaborative overview*" OR "systematic review*" OR "technology assessment*" OR "technology overview*" OR HTA OR HTAs OR "comparative efficacy" OR "comparative effectiveness" OR "outcomes research" OR "indirect comparison*" OR (("indirect treatment" OR mixed-treatment) AND comparison*) OR Embase* OR Cinahl* OR "systematic overview*" OR "methodological overview*" OR "methodologic overview*" OR "methodological review*" OR "methodologic review*" OR "quantitative review*" OR "quantitative overview*" OR "quantitative synthes*" OR "pooled analy*" OR Cochrane OR Medline OR Pubmed OR Medlars OR

handsearch* OR hand search* OR meta-regression* OR metaregression* OR “data synthes*” OR “data extraction” OR “data abstraction*” OR “mantel haenszel” OR peto OR der-simonian OR dersimonian OR “fixed effect*”)

ABI Inform Global

"shift work*" OR shiftwork* OR "evening shift*" OR "night shift*" OR "night-shift" OR "day shift*" OR "day-shift*" OR "night work*" OR "evening work*" OR "split shift*" OR "split-shift*" OR "rotating shift*" OR "rotating-shift*" OR "alternating work*" OR "alternating-work*" OR "non day work*" OR "non-day-work*" OR “work hours” OR “working hours” OR “overtime work” OR “extended work” OR “extended shift” OR “long work” OR “long working” OR “long shift” OR “12-hour shift” OR “12-hour work day” OR “compressed work” OR “four-day week” OR “compressed shift*” OR “rearranged work week” OR “shift length*” OR “shift duration” OR “long hours”

AND

"meta analy*" OR metanaly* OR metaanaly* OR "met analy*" OR "integrative research" OR "integrative review*" OR "integrative overview*" OR "research integration*" OR "research overview*" OR "collaborative review*" OR "collaborative overview*" OR "systematic review*" OR "technology assessment*" OR "technology overview*" OR HTA OR HTAs OR "comparative efficacy" OR "comparative effectiveness" OR "outcomes research" OR "indirect comparison*" OR (("indirect treatment" OR mixed-treatment) AND comparison*) OR Embase* OR Cinahl* OR "systematic overview*" OR "methodological overview*" OR "methodologic overview*" OR "methodological review*" OR "methodologic review*" OR "quantitative review*" OR "quantitative overview*" OR "quantitative synthes*" OR "pooled analy*" OR Cochrane OR Medline OR Pubmed OR Medlars OR handsearch* OR hand search* OR meta-regression* OR metaregression* OR "data synthes*" OR "data extraction" OR "data abstraction*" OR "mantel haenszel" OR peto OR der-simonian OR dersimonian OR "fixed effect*"

Business Source Premier

((DE "SHIFT systems" OR DE "NIGHT work" OR DE "COMPRESSED workweek" OR DE "FOUR day week") OR DE "WORKING hours" OR DE "EIGHT-hour movement" OR DE "FLEXIBLE work arrangements" OR DE "FLEXTIME" OR DE "THIRTY-five hour week" OR DE "THIRTY-hour week" OR DE "WORKWEEK")) OR TI ("shift work*" OR shiftwork* OR "evening shift*" OR "night shift*" OR "night-shift" OR "day shift*" OR "day-shift*" OR "night work*" OR "evening work*" OR "split shift*" OR "split-shift*" OR "rotating shift*" OR "rotating-shift*" OR "alternating work*" OR "alternating-work*" OR "non day work*" OR "non-day-work*") OR AU ("shift work*" OR shiftwork* OR "evening shift*" OR "night shift*" OR "night-shift" OR "day shift*" OR "day-shift*" OR "night work*" OR "evening work*" OR "split shift*" OR "split-shift*" OR "rotating shift*" OR "rotating-shift*" OR "alternating work*" OR "alternating-work*" OR "non day work*" OR "non-day-work*" OR “work hours” OR “working hours” OR “overtime work” OR “extended work” OR “extended shift” OR “long work” OR “long working” OR “long shift” OR “12-hour shift” OR “12-hour work day” OR “compressed work” OR “four-day week” OR “compressed shift*” OR “rearranged work week” OR “shift length*” OR “shift duration” OR “long hours”)

AND

TI ("meta analy*" OR metanaly* OR metaanaly* OR "met analy*" OR "integrative research" OR "integrative review*" OR "integrative overview*" OR "research integration*" OR "research overview*" OR "collaborative review*" OR "collaborative overview*" OR "systematic review*" OR "technology assessment*" OR "technology overview*" OR HTA OR HTAs OR "comparative efficacy" OR "comparative effectiveness" OR "outcomes research" OR "indirect comparison*" OR (("indirect treatment" OR mixed-treatment) AND comparison*) OR Embase* OR Cinahl* OR "systematic overview*" OR "methodological overview*" OR "methodologic overview*" OR "methodological review*" OR "methodologic review*" OR "quantitative review*" OR "quantitative overview*" OR "quantitative synthes*" OR "pooled analy*" OR Cochrane OR Medline OR Pubmed OR Medlars OR handsearch* OR hand search* OR meta-regression* OR metaregression* OR "data synthes*" OR "data extraction" OR "data abstraction*" OR "mantel haenszel" OR peto OR der-simonian OR dersimonian OR "fixed effect*") OR AB ("meta analy*" OR metanaly* OR metaanaly* OR "met analy*" OR "integrative research" OR "integrative review*" OR "integrative overview*" OR "research integration*" OR "research overview*" OR "collaborative review*" OR "collaborative overview*" OR "systematic review*" OR "technology assessment*" OR "technology overview*" OR HTA OR HTAs OR "comparative efficacy" OR "comparative effectiveness" OR "outcomes research" OR "indirect comparison*" OR (("indirect treatment" OR mixed-treatment) AND comparison*) OR Embase* OR Cinahl* OR "systematic overview*" OR "methodological overview*" OR "methodologic overview*" OR "methodological review*" OR "methodologic review*" OR "quantitative review*" OR "quantitative overview*" OR "quantitative synthes*" OR "pooled analy*" OR Cochrane OR Medline OR Pubmed OR Medlars OR handsearch* OR hand search* OR meta-regression* OR metaregression* OR "data synthes*" OR "data extraction" OR "data abstraction*" OR "mantel haenszel" OR peto OR der-simonian OR dersimonian OR "fixed effect*")

Agricultural & Environmental Science Database (including Risk Abstracts)

noft("shift work*" OR shiftwork* OR "evening shift*" OR "night shift*" OR "night-shift" OR "day shift*" OR "day-shift*" OR "night work*" OR "evening work*" OR "split shift*" OR "split-shift*" OR "rotating shift*" OR "rotating-shift*" OR "alternating work*" OR "alternating-work*" OR "non day work*" OR "non-day-work*" OR “work hours” OR “working hours” OR “overtime work” OR “extended work” OR “extended shift” OR “long work” OR “long working” OR “long shift” OR “12-hour shift” OR “12-hour work day” OR “compressed work” OR “four-day week” OR “compressed shift*” OR “rearranged work week” OR “shift length*” OR “shift duration” OR “long hours”) AND noft("meta analy*" OR metanaly* OR metaanaly* OR "met analy*" OR "systematic review*")

EconLit

"shift work*" OR shiftwork* OR "evening shift*" OR "night shift*" OR "night-shift" OR "day shift*" OR "day-shift*" OR "night work*" OR "evening work*" OR "split shift*" OR "split-shift*" OR "rotating shift*" OR "rotating-shift*" OR "alternating work*" OR "alternating-work*" OR "non day work*" OR "non-day-work*" OR “work hours” OR “working hours” OR “overtime work” OR “extended work” OR “extended shift” OR “long work” OR “long working” OR “long shift” OR “12-hour shift” OR “12-hour work day” OR “compressed work” OR “four-day week” OR “compressed shift*” OR “rearranged work week” OR “shift length*” OR “shift duration” OR “long hours”

AND

"meta analy*" OR metanaly* OR metaanaly* OR "met analy*" OR "integrative research" OR "integrative review*" OR "integrative overview*" OR "research integration*" OR "research overview*" OR "collaborative review*" OR "collaborative overview*" OR "systematic review*" OR "technology assessment*" OR "technology overview*" OR HTA OR HTAs OR "comparative efficacy" OR "comparative effectiveness" OR "outcomes research" OR "indirect comparison*" OR (("indirect treatment" OR mixed-treatment) AND comparison*) OR Embase* OR Cinahl* OR "systematic overview*" OR "methodological overview*" OR "methodologic overview*" OR "methodological review*" OR "methodologic review*" OR "quantitative review*" OR "quantitative overview*" OR "quantitative synthes*" OR "pooled analy*" OR Cochrane OR Medline OR Pubmed OR Medlars OR handsearch* OR hand search* OR meta-regression* OR metaregression* OR "data synthes*" OR "data extraction" OR "data abstraction*" OR "mantel haenszel" OR peto OR der-simonian OR dersimonian OR "fixed effect*"

ProQuest Dissertations

("shift work*" OR shiftwork* OR "evening shift*" OR "night shift*" OR "night-shift" OR "day shift*" OR "day-shift*" OR "night work*" OR "evening work*" OR "split shift*" OR "split-shift*" OR "rotating shift*" OR "rotating-shift*" OR "alternating work*" OR "alternating-work*" OR "non day work*" OR "non-day-work*" OR “work hours” OR “working hours” OR “overtime work” OR “extended work” OR “extended shift” OR “long work” OR “long working” OR “long shift” OR “12-hour shift” OR “12-hour work day” OR “compressed work” OR “four-day week” OR “compressed shift*” OR “rearranged work week” OR “shift length*” OR “shift duration” OR “long hours”)

AND

("meta analy*" OR metanaly* OR metaanaly* OR "met analy*" OR "integrative research" OR "integrative review*" OR "integrative overview*" OR "research integration*" OR "research overview*" OR "collaborative review*" OR "collaborative overview*" OR "systematic review*" OR "technology assessment*" OR "technology overview*" OR HTA OR HTAs OR "comparative efficacy" OR "comparative effectiveness" OR "outcomes research" OR "indirect comparison*" OR (("indirect treatment" OR mixed-treatment) AND comparison*) OR Embase* OR Cinahl* OR "systematic overview*" OR "methodological overview*" OR "methodologic overview*" OR "methodological review*" OR "methodologic review*" OR "quantitative review*" OR "quantitative overview*" OR "quantitative synthes*" OR "pooled analy*" OR Cochrane OR Medline OR Pubmed OR Medlars OR handsearch* OR hand search* OR meta-regression* OR metaregression* OR "data synthes*" OR "data extraction" OR "data abstraction*" OR "mantel haenszel" OR peto OR der-simonian OR dersimonian OR "fixed effect*")

Epistemonikos

"shift work*" OR shiftwork* OR "evening shift*" OR "night shift*" OR "night-shift" OR "day shift*" OR "day-shift*" OR "night work*" OR "evening work*" OR "split shift*" OR "split-shift*" OR "rotating shift*" OR "rotating-shift*" OR "alternating work*" OR "alternating-work*" OR "non day work*" OR "non-day-work*" OR “work hours” OR “working hours” OR “overtime work” OR “extended work” OR “extended shift” OR

“long work” OR “long working” OR “long shift” OR “12-hour shift” OR “12-hour work day” OR “compressed work” OR “four-day week” OR “compressed shift*” OR “rearranged work week” OR “shift length*” OR “shift duration” OR “long hours” [Filters: protocol=no, classification=systematic-review]

Prospero

“shift work*” OR shiftwork* OR “evening shift*” OR “night shift*” OR “night-shift” OR “day shift*” OR “day-shift*” OR “night work*” OR “evening work*” OR “split shift*” OR “split-shift*” OR “rotating shift*” OR “rotating-shift*” OR “alternating work*” OR “alternating-work*” OR “non day work*” OR “non-day-work*” OR “work hours” OR “working hours” OR “overtime work” OR “extended work” OR “extended shift” OR “long work” OR “long working” OR “long shift” OR “12-hour shift” OR “12-hour work day” OR “compressed work” OR “four-day week” OR “compressed shift*” OR “rearranged work week” OR “shift length*” OR “shift duration” OR “long hours”

Cochrane Database of Systematic Reviews (includes Cochrane protocols)

“shift work*” OR shiftwork* OR “evening shift*” OR “night shift*” OR “night-shift” OR “day shift*” OR “day-shift*” OR “night work*” OR “evening work*” OR “split shift*” OR “split-shift*” OR “rotating shift*” OR “rotating-shift*” OR “alternating work*” OR “alternating-work*” OR “non day work*” OR “non-day-work*” OR “working hours” OR “overtime work” OR “extended work” OR “extended shift” OR “long work” OR “long working” OR “long shift” OR “12 hour shift” OR “12 hour work day” OR “compressed work” OR “four-day week” OR “compressed shift*” OR “rearranged work week” OR “shift length*” OR “shift duration” OR “long hours”

List of Grey Literature Sources – searched Jan 2019/not updated in April 2019

US: Grey Lit
http://www.greylit.org/library/search#wt=json&facet=true&q=shift%20work&q.op=AND&fl=id&qt=dismax&sort=created%20desc&page=1&per_page=10&start=0&qf=full_text&facet.field=publisher&facet.field=full_subjects

Europe: Open Grey
http://www.opengrey.eu/search/request?q=keyword%3A%28Travail+post%C3%A9%29&b=0

CADTH: Canada
https://www.cadth.ca/search?keywords=shift+work

SRDR (ARHQ): Sys Review Depository - Zero results (11 Jan 2019)

CDC – zero results (11 Jan 2019)

Working Time Society
http://www.workingtime.org/archive
