# Supplementary material for: Shift work and long work hours and their association with chronic health conditions: A systematic review of systematic reviews with meta-analyses
Source: PLoS One. 2020 Apr 2;15(4):e0231037. doi: 10.1371/journal.pone.0231037 (PMC7117719; doi:10.1371/journal.pone.0231037)

S4. Main Pooled Results of Included Reviews Per Condition

I. Shift work

A. All-cause mortality

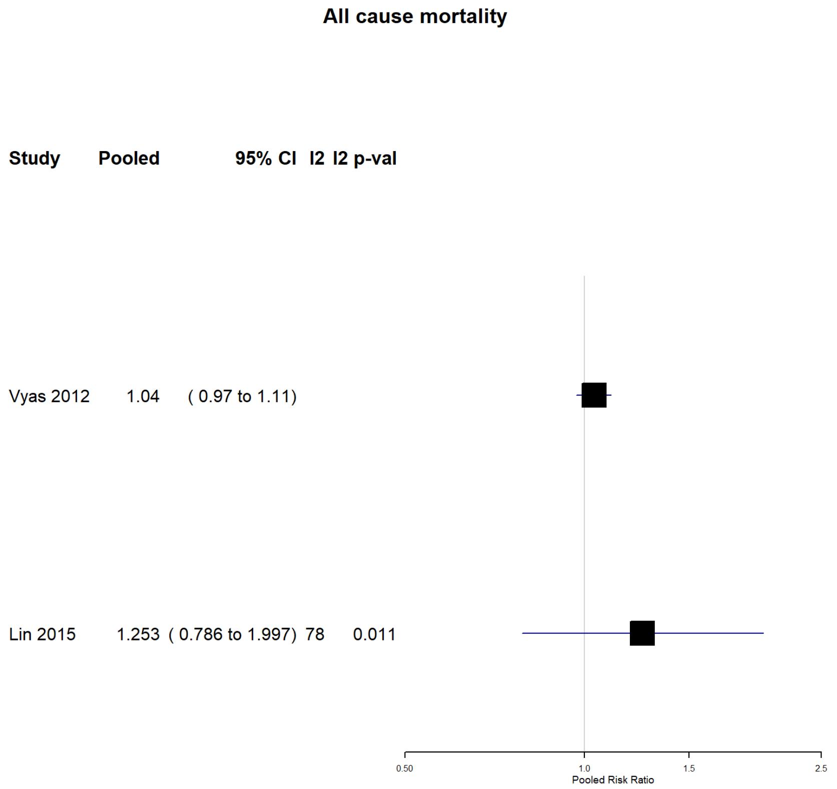

B. Cancers

a. Breast Cancer

Breast cancer risk

Study	Pooled	95% CI	I2	I2 p-val
Travis 2016	0.99	(0.95 to 1.03)		0.52
Wang 2013	1.19	(1.05 to 1.35)	79.5	0
Megdal 2005	1.48	(1.36 to 1.61)		0.62
Kamdar 2013	1.21	(1 to 1.47)	75.8	0
Jia 2013	1.2	(1.08 to 1.33)	63.4	0
He 2015	1.19	(1.08 to 1.32)	76.1	0
Erren 2008	1.4	(1.3 to 1.6)		0.18
Liu 2018	1.22	(1.08 to 1.38)	81.2	0.001
Li 2017	1.1	(1.03 to 1.18)	62	0.01

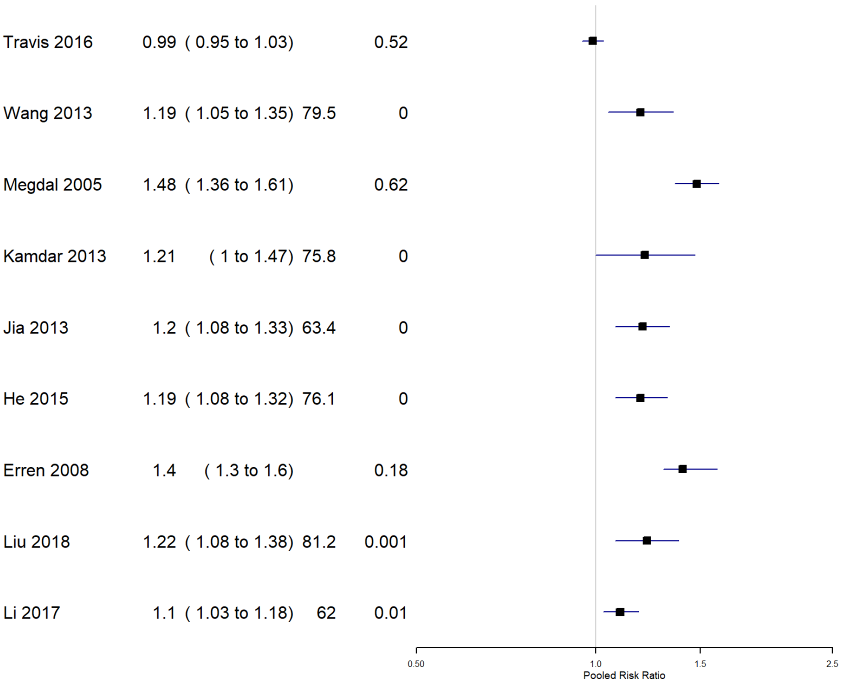

b. Prostate Cancer

Prostate cancer risk

Study	Pooled	95% CI	I2	I2 p-val
Du 2017	1.05	(1 to 1.11)	24	0.23
Liu 2018	1.26	(1.05 to 1.52)	73.2	0.001

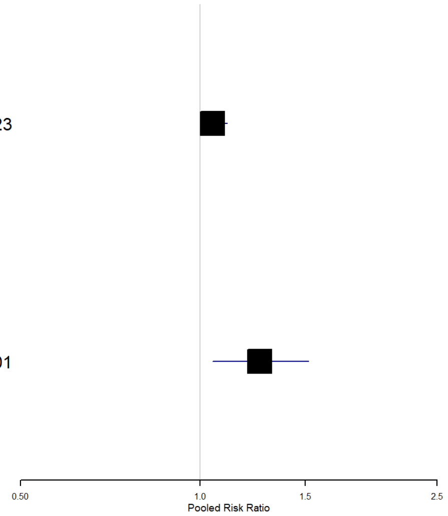

c. Other Cancers

Others Cancers

Study	Shift type	Outcome	Pooled	95% CI	I2	I2 p-val
Wang 2015	shift work (any type)	Colorectal cancer	1.318	(1.21 to 1.55)	77.7	0
Liu 2018	shift work (any type)	Lung cancer risk	1.08	(0.87 to 1.35)	53.4	0.073
Liu 2018	shift work (any type)	Reproductive system cancer risk	1.06	(0.85 to 1.32)	49.5	0.078
Liu 2018	shift work (any type)	Digestive system cancer risk	1.15	(1.01 to 1.32)	40.2	0.081
Liu 2018	shift work (any type)	Hematologic system cancer risk	1.08	(0.99 to 1.17)	54.7	0.066
Liu 2018	shift work (any type)	Skin cancer risk	0.93	(0.5 to 1.74)	74.9	0.019

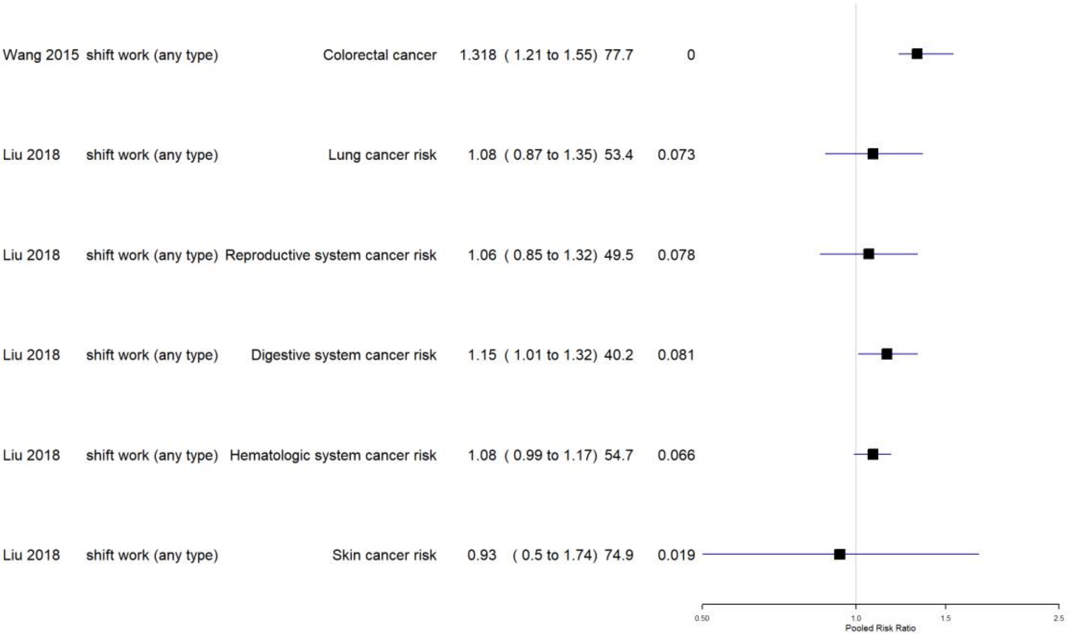

C. Cardiovascular Diseases

CVD risk

Study	Outcome	Pooled	95% CI	I2	I2 p-val
Torquati 2018	Cardiovascular disease risk	1.17	(1.09 to 1.25)	67	0
Vyas 2012	Cardiovascular events	1.24	(0.81 to 1.89)		
Vyas 2012	Cardiovascular mortality	1.14	(0.98 to 1.32)		
Lin 2015	Cardiovascular Mortality	1.027	(1.001 to 1.053)	0	0.806
Vyas 2012	Cerebrovascular mortality	1.12	(0.89 to 1.4)		
Torquati 2018	CHD morbidity	1.26	(1.1 to 1.43)	58.2	0.003
Vyas 2012	Coronary Event	1.24	(1.1 to 1.39)	85	
Vyas 2012	Coronary mortality	1.08	(0.97 to 1.21)		
Torquati 2018	CVD mortality	1.13	(0.99 to 1.29)	55.6	0.004
Cheng 2019	Ischemic heart disease risk	1.13	(1.08 to 1.2)	52.7	0
Vyas 2012	Ischemic stroke	1.05	(1.01 to 1.09)	0	
Vyas 2012	Myocardial infarction	1.23	(1.15 to 1.31)	0	
Cheng 2019	Myocardial infarction	1.27	(1.17 to 1.39)	0	0.643
Torquati 2018	Other CVD morbidity	1.04	(0.96 to 1.14)	48	0.104
Li 2016	Stroke risk	1.03	(1 to 1.07)	0	0.496

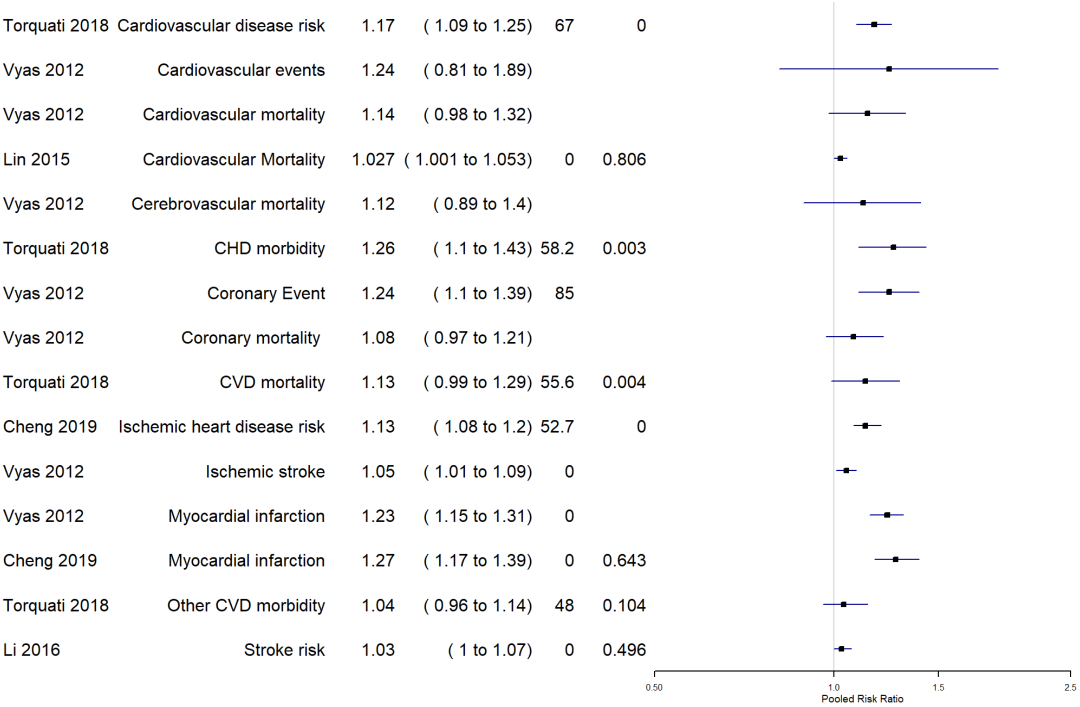

Note: CHD – coronary heart disease, CVD – cardiovascular disease

D. Complications of Pregnancy

Complications of Pregnancy

Study	Outcome	Shift work type	Pooled	95% CI	I2	I2 p-val
Cai 2019	Gestational hypertension	rotating shift work	1.19	(1.1 to 1.29)	0	
Cai 2019	Gestational hypertension	fixed night shift work	1.19	(0.97 to 1.45)	2	
Bonzini 2011	Low Birthweight	shift work (any type)	1.27	(0.93 to 1.74)		0.39
Cai 2019	Low Birthweight	rotating shift work	1.41	(0.82 to 2.41)	20	
Cai 2019	Low Birthweight	fixed night shift work	1.44	(0.76 to 2.75)	0	
Bonde 2013	Miscarriage	shift work (any type)	1.12	(0.96 to 1.3)		0.53
Cai 2019	Miscarriage	rotating shift work	1.05	(0.85 to 1.29)	64	
Cai 2019	Miscarriage	fixed night shift work	1.23	(1.03 to 1.47)	33	
Cai 2019	preeclampsia	rotating shift work	1.75	(1.01 to 3.01)	75	
Cai 2019	preeclampsia	fixed night shift work	1.05	(0.63 to 1.75)	0	
Palmer 2013	preterm birth	shift work (any type)	1.14	(1.01 to 1.3)	47.5	0.012
Mozurkewich 2000	preterm birth	shift work (any type)	1.24	(1.06 to 1.46)		
Bonzini 2011	preterm birth	shift work (any type)	1.16	(1 to 1.33)		0.006
Bonzini 2007	preterm birth	shift work (any type)	1.2	(1.01 to 1.42)		0.002
Cai 2019	preterm birth	rotating shift work	1.13	(1 to 2.81)	31	
Cai 2019	preterm birth	fixed night shift work	1.21	(1.03 to 1.42)	36	
Palmer 2013	Small for gestational age (SGA)	shift work (any type)	1.01	(0.92 to 1.1)		
Bonzini 2011	Small for gestational age (SGA)	shift work (any type)	1.12	(1.03 to 1.22)		0.39
Bonzini 2007	Small for gestational age (SGA)	shift work (any type)	1.07	(0.96 to 1.19)	3.3	0.51
Cai 2019	Small for gestational age (SGA)	rotating shift work	1.18	(1.01 to 1.38)	0	
Cai 2019	Small for gestational age (SGA)	fixed night shift work	1.08	(0.86 to 1.35)	0	
Quansah 2010	Spontaneous Abortion	shift work (any type)	1.44	(1.06 to 1.95)	61.9	0.049

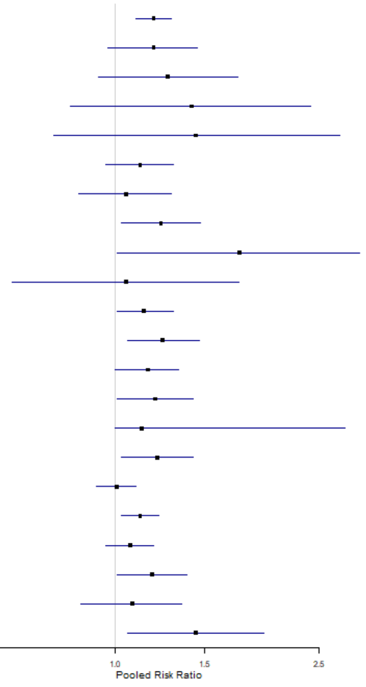

E. Depression

Depression risk

Study	Pooled	95% CI	I2	I2 p-val
Lee 2017	1.43	(1.24 to 1.64)	78	
Angerer 2017	1.42	(0.92 to 2.19)	74.4	

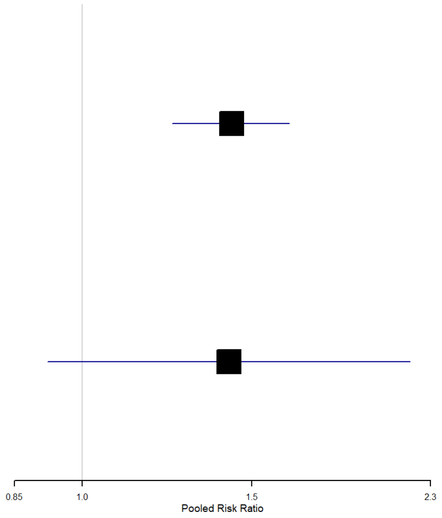

F. Diabetes mellitus

Diabetes risk

Study	Pooled	95% CI	I2	I2 p-val
Anothaisintawee 2016	1.4	(1.18 to 1.66)	95	0
Gan 2015	1.09	(1.05 to 1.12)	40.9	0.014

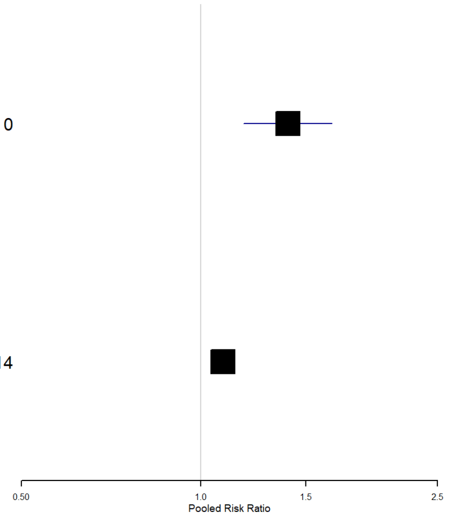

G. Hypertension

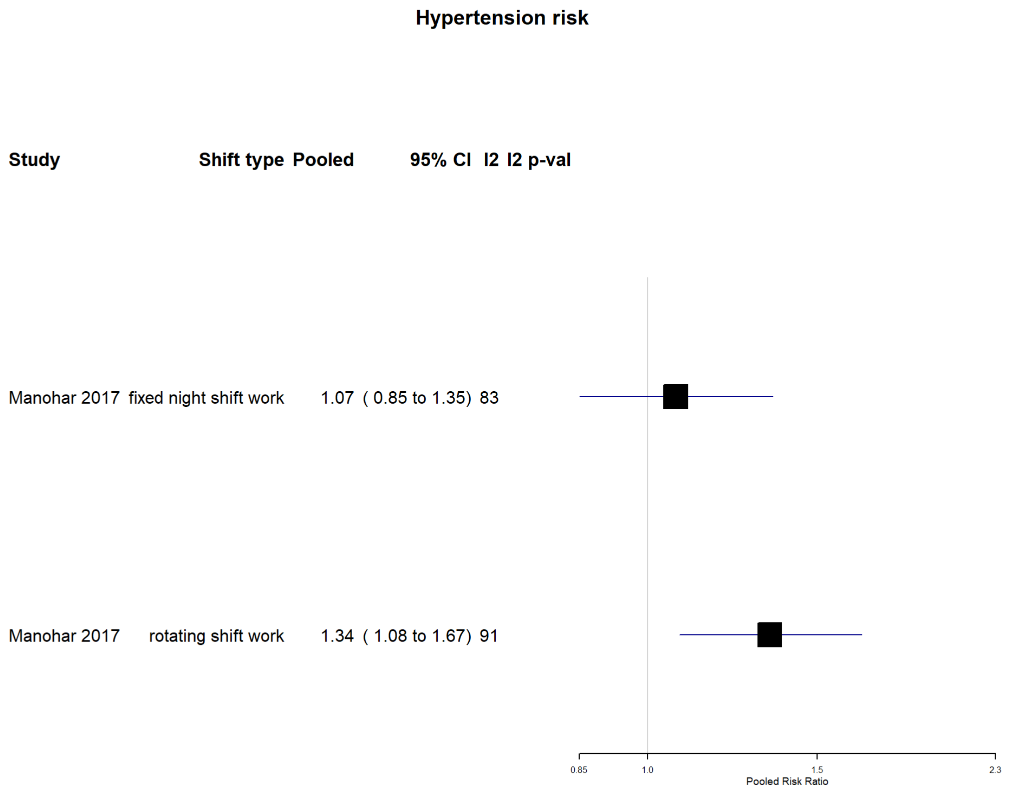

H. Metabolic syndrome

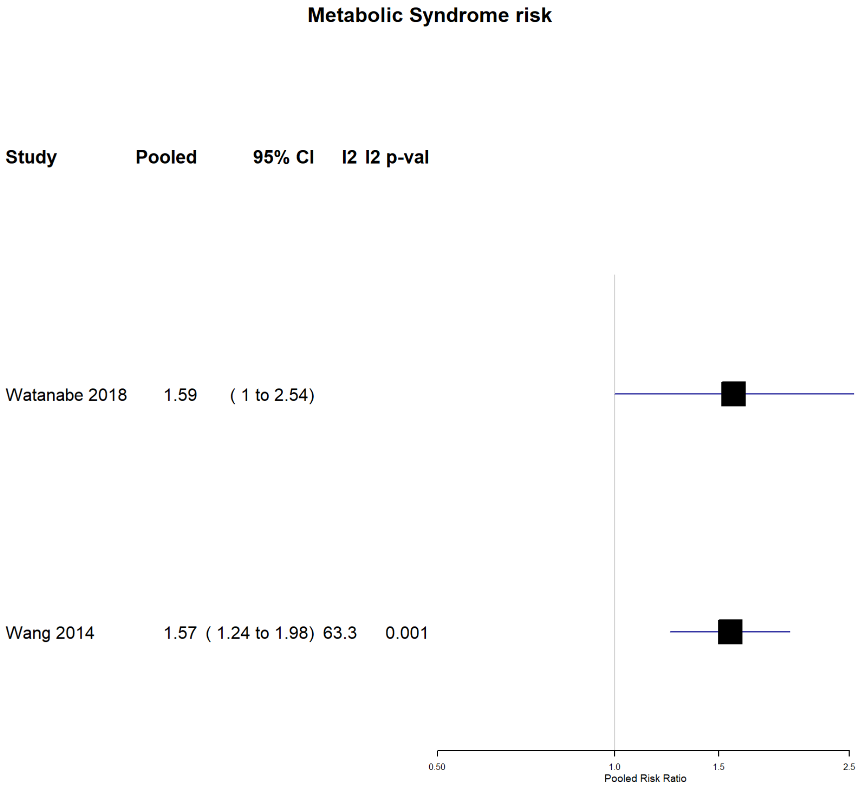

I. Obesity

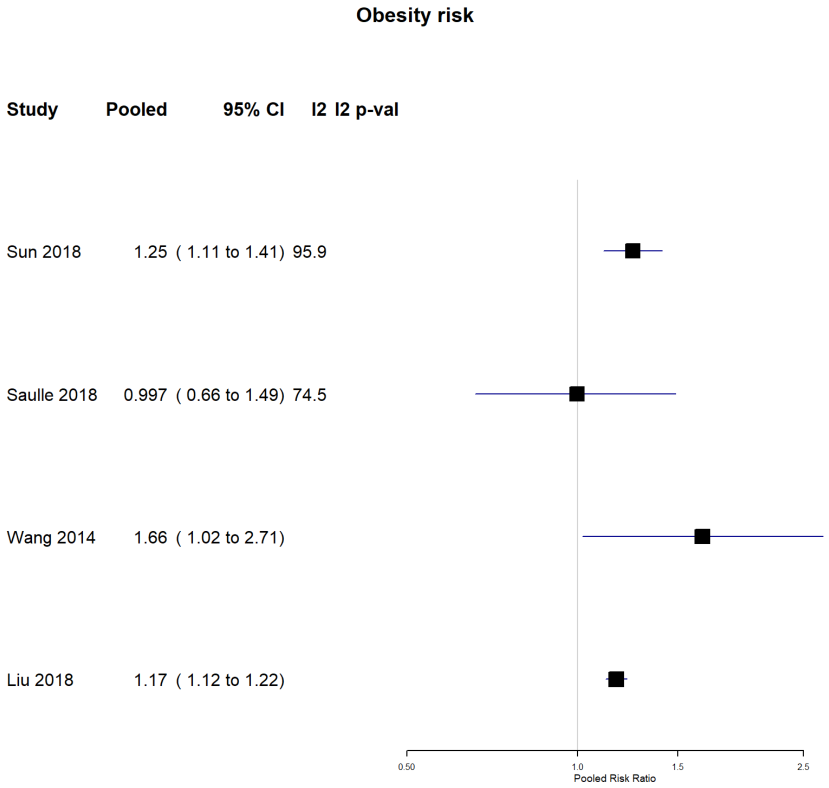
Obesity risk
Study Pooled 95% CI I2 I2 p-val
Sun 2018 1.25 (1.11 to 1.41) 95.9
Saulle 2018 0.997 (0.66 to 1.49) 74.5
Wang 2014 1.66 (1.02 to 2.71)
Liu 2018 1.17 (1.12 to 1.22)
0.50
1.0
1.5
2.5
Pooled Risk Ratio

J. Occupational injuries

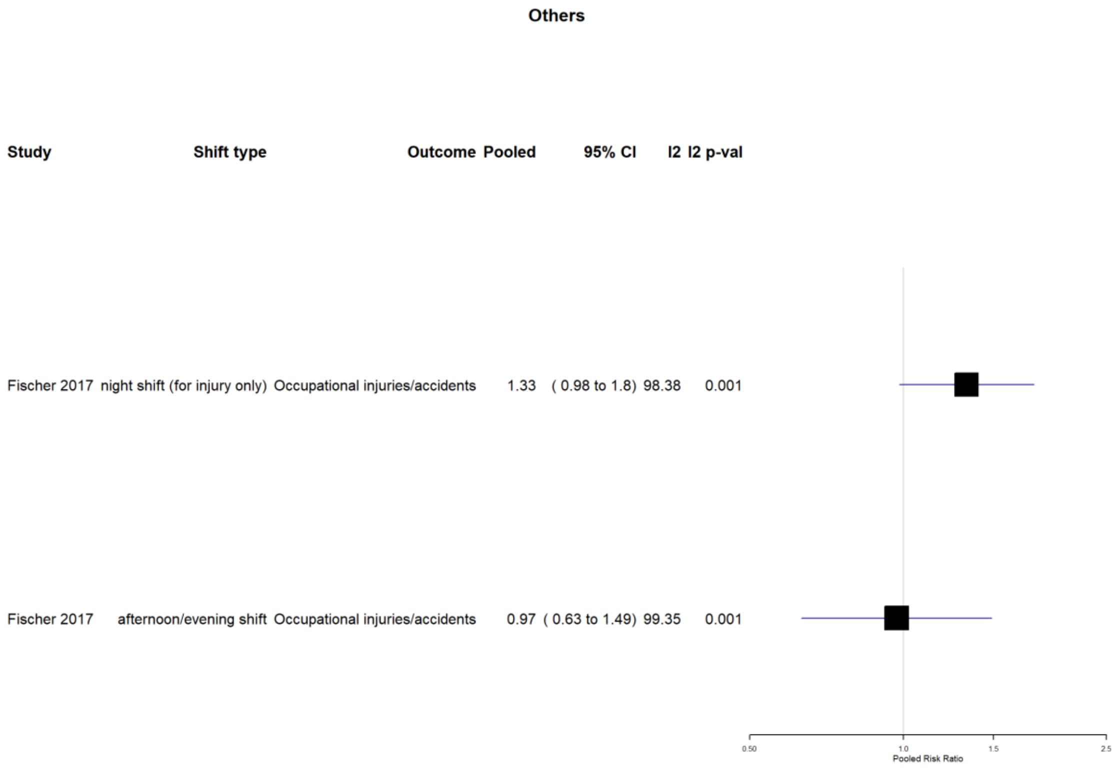

III. Long work hours

A. Cardiovascular diseases

CVD risk

Study	Outcome	Pooled	95% CI	I2	I2 p-val
Kivimaki 2015	Stroke risk	1.33	(1.11 to 1.61)	0	0.67
Kivimaki 2015	Coronary heart disease	1.13	(1.02 to 1.26)	0	0.49
Kang 2012	Cardiovascular disease	1.37	(1.11 to 1.7)		0.037
Virtanen 2012	Coronary heart disease	1.8	(1.42 to 2.29)	61.9	0.002

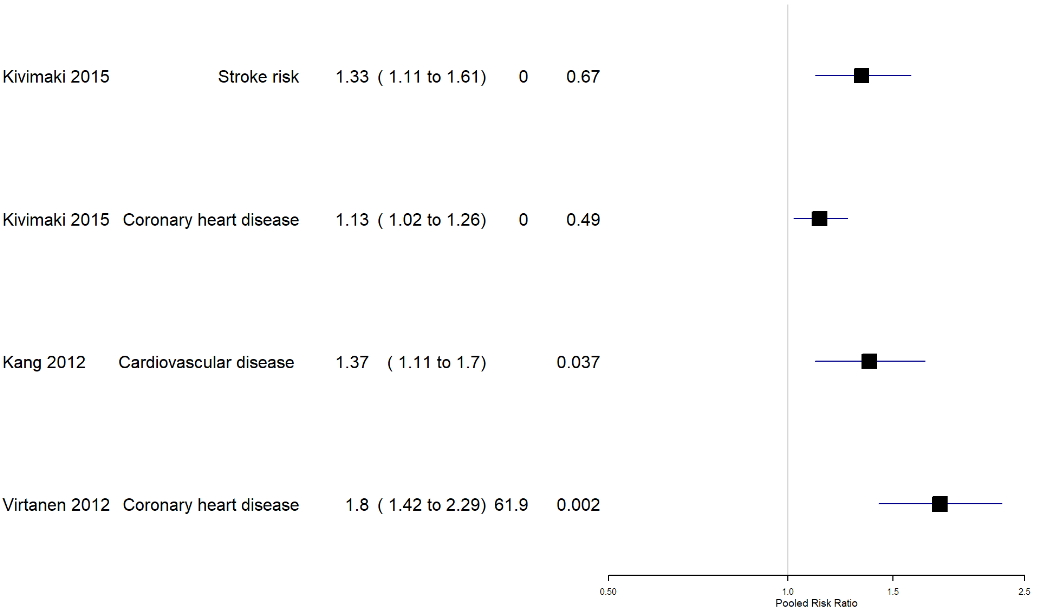

B. Complications of Pregnancy

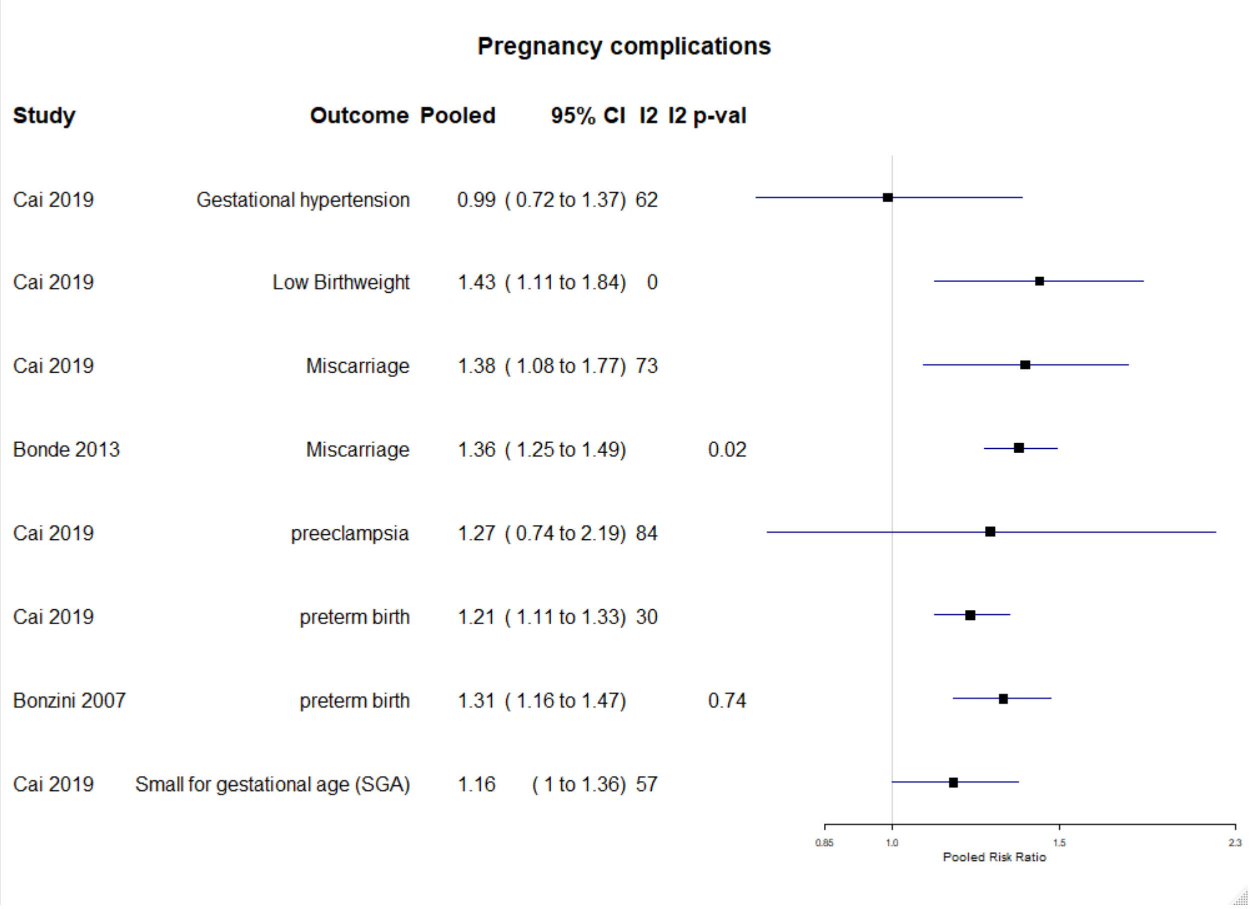

C. Diabetes mellitus

Diabetes risk

Study	Pooled	95% CI	I2	I2 p-val
Kivimaki 2015	1.18	(0.6 to 2.32)		0.0065
Cosgrove 2012	1.14	(0.35 to 3.72)	67	

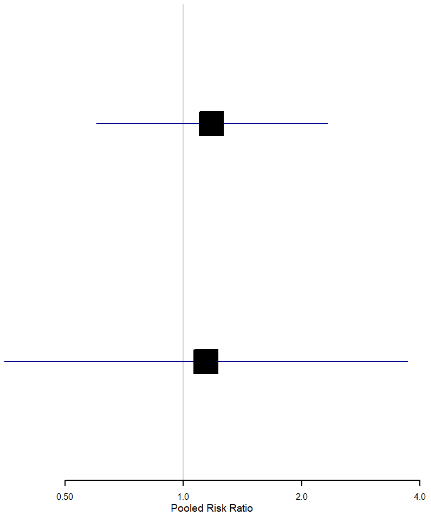

D. Depression

Depression risk

Study	Pooled	95% CI	I2	I2 p-val
Watanabe 2016	1.08	(0.83 to 1.39)	16.7	0.294
Virtanen 2018	1.14	(1.03 to 1.25)	45.1	0.004

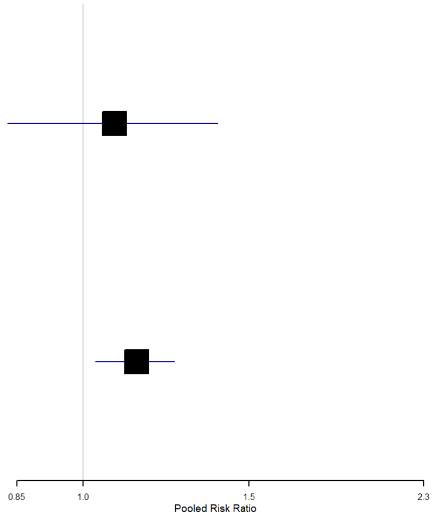

Supplement: S4 File — (PDF) [file pone.0231037.s004.pdf]
