# Supplementary material for: Shift work and long work hours and their association with chronic health conditions: A systematic review of systematic reviews with meta-analyses
Source: PLoS One. 2020 Apr 2;15(4):e0231037. doi: 10.1371/journal.pone.0231037 (PMC7117719; doi:10.1371/journal.pone.0231037)
Supplement: S5 File — (PDF) [file pone.0231037.s005.pdf]

Assessing the risk for chronic and high cost conditions due to shift work and long working hours: an umbrella review

Ado Rivera, Megan McHugh, Linda O'Dwyer, Maxwell Akanbi

Citation

Ado Rivera, Megan McHugh, Linda O'Dwyer, Maxwell Akanbi. Assessing the risk for chronic and high cost conditions due to shift work and long working hours: an umbrella review. PROSPERO 2019 CRD42019122084 Available from:
https://www.crd.york.ac.uk/prospero/display_record.php?ID=CRD42019122084

Review question

Among employees of any industry, how does shift work and long work hours affect risk of acquiring chronic or high cost conditions?

Which chronic or high cost conditions have established associations with shift work and long work hours?

What is the magnitude of associated risk with chronic conditions and shift work and long work hours?

Searches

We will search the following electronic bibliographic databases: MEDLINE, Embase, Scopus, CINAHL, Web of Science, PsycINFO, ABI Inform Global, Business Source Premier, and Risk Abstracts. We will look for grey literature in the following sites: Grey Literature Report, OpenGrey, CADTH, Systematic review repository (AHRQ), Epistomonikos, PROSPERO, Working time conference meetings/abstracts, CDC National Institute for Occupational Safety and Health, Proquest Dissertations

We will also search references for included studies and contact a small number of experts to identify additional studies.

There will be no language, country or date restrictions in the search but we will only include articles written in English, Spanish or French.

Types of study to be included

We will include any review that (1) performed a systematic search (as opposed to purposive or undocumented selection of articles) of literature using at least one database, (2) exposure is shift work or long work hours, (3) adult human population, (4) outcome is a chronic condition or identified high cost condition, (5) one of the objectives or questions of the review was to report quantitative association of a chronic or high cost condition and shift work OR review focused on quantitative studies and (6) performed a meta-analysis of risk association measures. Studies that focus on mechanisms, risk factors, biomarkers, quality of life, or employee outcomes will be excluded. Studies that focus on interventions for shift workers will also be excluded.

Condition or domain being studied

Chronic conditions and diseases, following CDC's definition, are "conditions that last 1 year or more and require ongoing medical attention or limit activities of daily living or both." Examples of these are type 2 diabetes, obesity, sleep disorders, and substance abuse.

Selected high cost conditions that were either part of nine most prevalent conditions that lead to health expenditure identified in the 2013 Medical Expenditure Panel Survey or belong to the top 20 causes of United States personal healthcare spending data from 1996 to 2013. Example conditions are traumatic injuries, diabetes, hypertension, pain-related conditions, pregnancy, and lower respiratory tract infections.

Participants/population

Human Adults (18 years or older) working in any industry.

Intervention(s), exposure(s)
Shift work (all types such as night work and rotating night work) and long work hours (>8 hours per day or >40 hours per week).

Comparator(s)/control
No shift work (shift work) or 8 hour day shift (long work hours).

Context

Main outcome(s)
Risk of chronic or high cost condition comparing shift work employees to non-shift work employees measured using Odds ratio, Risk ratio or Hazards ratio.

Timing and effect measures
Pooled risk or association measures (e.g. OR, RR, HR, effect size).

Additional outcome(s)
None.

Timing and effect measures
Not applicable.

Data extraction (selection and coding)
Two reviewers will independently screen titles and abstracts, and later, full-text articles. Studies will be discarded only if both reviewers agree. Discrepancies will be resolved through conversation and/or use of a third reviewer.

A standardised, pre-piloted form will be used to extract data from the included studies. Extracted information will include: review objectives/questions, population, chronic conditions, shift work types and definitions, inclusive years of search, databases searched, number (total, types, countries of origin) of included studies, publication date range of included studies, instrument(s) used for quality assessment, summary of quality assessment of included studies, narrative summary of key findings, method of pooling used, type(s) of association/measure used, total number of participants for pooled measure, and pooled risk/association including subgroup analysis that are deemed relevant by the study team.

Two review authors will extract data independently, discrepancies will be identified and resolved through discussion (with a third author where necessary). Missing data deemed important by the reviewers will be requested from study authors.

Risk of bias (quality) assessment
Reviews will be assessed using either the AMSTAR version 2. Assessment will be done independently by two reviewers with discrepancies resolved through discussion (or a third reviewer).

Strategy for data synthesis
A summary table listing chronic conditions and the synthesized findings on its association with shift work will be included. Pooled measures of association from the high quality reviews may be included in this table. We will also comment on the gaps in the literature or noted weaknesses of the included reviews. The overall evidence will be assessed using the GRADE framework.

Analysis of subgroups or subsets
Findings of high quality reviews compared to the overall findings including all reviews. We will also compare findings according to study design (case-control only vs cohort), age, gender, and race, if available.

Contact details for further information
Ado Rivera
adovichrivera2021@u.northwestern.edu

Organisational affiliation of the review
Northwestern University

Review team members and their organisational affiliations

Mr Ado Rivera. Northwestern University
Assistant/Associate Professor Megan McHugh. Northwestern University
Miss Linda O'Dwyer. Northwestern University
Dr Maxwell Akanbi. Northwestern University

Type and method of review

Epidemiologic, Review of reviews, Systematic review

Anticipated or actual start date

01 January 2019

Anticipated completion date

30 August 2019

Funding sources/sponsors

Robert Wood Johnson Foundation

Conflicts of interest

Language

English

Country

United States of America

Stage of review

Review Ongoing

Subject index terms status

Subject indexing assigned by CRD

Subject index terms

Costs and Cost Analysis; Humans; Risk

Date of registration in PROSPERO

20 February 2019

Date of publication of this version

19 June 2019

Details of any existing review of the same topic by the same authors

Stage of review at time of this submission

Stage	Started	Completed
Preliminary searches	Yes	Yes
Piloting of the study selection process	Yes	Yes
Formal screening of search results against eligibility criteria	Yes	No
Data extraction	Yes	No
Risk of bias (quality) assessment	No	No
Data analysis	No	No

Versions

20 February 2019
25 March 2019
19 June 2019

PROSPERO
This information has been provided by the named contact for this review. CRD has accepted this information in good faith and registered the review in PROSPERO. The registrant confirms that the information supplied for this submission is accurate and complete. CRD bears no responsibility or liability for the content of this registration record, any associated files or external websites.
